# The Outcomes of Organizational Citizenship Behaviors in Part-Time and Temporary Working University Students

**DOI:** 10.3390/bs13080697

**Published:** 2023-08-21

**Authors:** Emma Johansson, Rona Hart

**Affiliations:** School of Psychology, University of Sussex, Falmer, Brighton BN1 9RH, UK; esmj20@sussex.ac.uk

**Keywords:** Organizational Citizenship Behavior (OCB), part-time employment, prosocial behavior

## Abstract

The personal outcomes of Organizational Citizenship Behavior (OCB) have recently gained popularity in research, but it is rarely studied in part-time or temporary employees and, in particular, in employed university students. The aim of the current study was to address this gap in the literature by investigating the outcomes of OCB, including job stress, work–university conflict, work–leisure conflict, intent to quit, well-being, and job satisfaction, in university students who undertake contingent and part-time work. Using a correlational research design, data collection was conducted through an online survey administered to 122 employed university students. The correlation analysis revealed that OCB correlated positively with work–university conflict and work–leisure conflict, which is aligned with earlier work. However, in contrast to earlier findings, OCB did not correlate with well-being, stress, job satisfaction or intent to quit. Regression analyses revealed that OCB positively predicted job satisfaction, when entered together with work–university conflict, job stress, and intent to quit. OCB also predicted job stress when entered with job satisfaction. However, OCB did not predict well-being. In turn, work–university conflict negatively predicted well-being. The current findings differ from the wider literature on full-time employees, which suggests a need for further research to examine why these differences exist and what are their practical implications.

## 1. Introduction

Employees commonly help each other in workplaces and support their organization in a manner that extends beyond their formal job requirements, including, for example, helping a new employee, sharing resources, taking on additional tasks, advising a colleague or covering for someone’s absence. This type of behavior is termed Organizational Citizenship Behavior (OCB) [1,2]. Research has historically focused on the antecedents and positive personal outcomes of these behaviors, and fewer studies examined the negative consequences of OCBs, though this is a topic that is currently receiving increased attention [3,4,5,6,7]. However, research concerning OCB in part-time or in contingent employees is scarce, and to our knowledge there is only one qualitative study and no quantitative research on part-time or temporary employees who are also university students. The current study aimed to address this gap in the literature by examining the consequences of OCB in university students who work on temporary contracts and part time. Using a quantitative correlational methodology, the study examined the degree to which these employees display OCBs and the associations between these behaviors and their well-being, job satisfaction, job stress, work–university conflict, work–leisure conflict, and intent to quit.

In what follows, we review the literature on OCB, examine the scarce literature on OCB in part-time and on contingent employees, offer some background and context on employed university students, and present the current study and its hypotheses. Next, we detail the methods applied in this study and the measures used, present the research findings, and discuss the findings in relation to the extent literature. We conclude with the contribution of the paper, its limitations, and future directions.

We note that due to the scarcity of research on OCB in part-time or temporary employees, the review provided below mainly draws on the literature on OCB in full-time employees in order to offer a sound theoretical footing and context for the study, as well as to enable the deduction of hypotheses.

### 1.1. OCB Review

OCB was originally defined as behavior that promotes effective functioning of organizations but is not directly required of an employee by their employer [1,8]. It has since been redefined to include any nonmandatory work-related behaviors that exceed what is formally required by the employer, which supports the social or psychological environment of an organization [2]. OCB is therefore considered a type of prosocial behavior that occurs in the work domain. As such, it fits within the larger umbrella term of “prosociality” [9] (p. 9), defined as “an umbrella term that encompasses dispositions, voluntary behaviors and processes that are focused on or contribute to the welfare of others.”

According to Organ and his colleagues [1,2,10], OCB consists of five components:

- Altruism: This is the willingness to help others within the organization without expecting anything in return. Altruistic behavior can range from assisting colleagues with tasks to offering guidance to new employees.
- Conscientiousness: This refers to the inclination of employees to go above and beyond the basic requirements of their job, such as being punctual, working extra hours, or following company rules even when not monitored.
- Sportsmanship: This component of OCB refers to an employee’s ability to tolerate less-than-ideal circumstances without complaining or causing disruptions, including accepting decisions made by management, and dealing with workplace issues positively.
- Courtesy: This theme involves proactive behaviors that help prevent work-related conflicts, such as communicating effectively with colleagues, or alerting them to changes that may affect their work.
- Civic virtue: This aspect of OCB refers to behaviors that show responsible and constructive involvement in the political life of the organization. It includes participation in meetings, engagement with organizational policies, and expressing opinions for the benefit of the organization.

Recent literature suggested the addition of other dimensions to OCB including:

- Voice behavior: An employee’s constructive challenge to the status quo [11].
- Ecological behavior: Voluntarily doing more than the job requires to promote or protect the environment [12].

Williams and Anderson [13] organized these components into two categories:

- OCBI: Behaviors directed toward other individuals with the aims of benefitting them.
- OCBO: Behaviors directed toward the organization with the aim of contributing to the organization.

As noted earlier, until recently, much of the research on OCB has focused on antecedents of OCB, and the positive outcomes of OCB mainly for the employees, with less research looking into the outcomes of OCB for the organization, and the negative outcomes of OCB for both individuals and organizations [3,6]. Below, a brief review of the literature is offered.

### 1.2. The Benefits of OCB

Research on OCB has documented the variety of benefits that can be gained from OCB both for the individuals involved as well as for the organization. The key benefits for individual employees include:

- Improved well-being: Employees who engage in OCB often experience better psychological well-being [14,15] and happiness [16]. Similar findings relating to employee well-being have been reported both in individualistic and collectivistic cultures (see for example research on Chinese employees [17], Indian [15], Spanish [14], and American samples [18]).
- Greater job satisfaction: Employees who engage in helping behavior tend to feel more satisfied with their jobs [19,20,21]. However, we note that job satisfaction has also been examined as an antecedent of OCB, a point that we shall explore below.
- Improved performance and performance evaluations: OCB is associated with improved performance [11,22,23]. In turn, employees who exhibit OCBs are often viewed more positively by their superiors, which can lead to better performance appraisals, favorable reward allocation decisions [24,25,26,27], and potentially promotions [26]. Ng and Feldman [28] argued that OCB can be seen as a form of investment in one’s career, increasing the chances of promotions and pay raises.
- Enhanced peer relations and cooperation: Engaging in OCB can improve relationships with coworkers [29], reduce conflict [30], promote cooperative behaviors such as knowledge sharing [31,32], and it can also create social capital for employees [33].
- Reduced stress: OCB has been found to negatively correlate with job stress [34]. However, as we shall demonstrate below, this finding has not been consistent in the literature, and some contradictory findings have emerged.
- Reduced counterproductive behaviors: In a meta-analysis on the association between OBC and counterproductive work behaviors, Dalal [35] reported on a negative but modest correlation. However, there are contradictory findings on this point which we explore in more detail below.

The key benefits of OCB for organizations include:

- Increased organizational effectiveness: OCB has been found to lead to improved productivity, efficiency, cost reduction, and profitability of the organization [29,36,37,38,39,40,41].
- Improved team performance: OCB promotes positive interpersonal relationships, leading to better team dynamics, team cohesion, and improved team performance [42,43,44].
- Reduced employee turnover: Organizations with high levels of OCBs tend to experience lower rates of employee turnover [45,46].
- Enhanced customer satisfaction: OCB have been found to contribute to increased customer satisfaction and loyalty [47].
- Favorable organizational reputation: When OCB is common in the workplace, it makes the organization a more attractive place to work for [10], hence improving its public image and reputation.

### 1.3. The Antecedents of OCB

Research on OCB has documented the variety of antecedents that can prompt the occurrence of OCB. Podsakoff et al. [38] classified the common antecedents into four categories: employee characteristics, organizational features, task characteristics, and leadership behaviors. These include:

- Organizational justice: Employees’ perceptions of fairness and ethics within their organization have been found to drive OCB [48,49,50,51]. Accordingly, organizational politics were reported to be negatively correlated with OCB [52].
- Organizational commitment: Employees who are committed to their organizations tend to show higher levels of OCB [53]. However, as we shall demonstrate below, there are some inconsistent findings around the association between OCB and intent to quit.
- Organizational identification: In an extensive systematic review, Sidorenkov et al. [54] reported on a positive moderate association between identification and OCB.
- Job involvement: Similar to commitment, job involvement has been shown to promote OCB [55,56].
- Job satisfaction: As mentioned earlier, while performing OCB can lead to increased job satisfaction, it is also considered an antecedent to OCB, suggesting that employees who are satisfied with their jobs are more likely to go above and beyond their formal job responsibilities to contribute their time and resources to others [38,57,58].
- Social environment/relationships at work: The behavior of leaders and employees’ relationships with the leader can significantly influence OCB [38,42]. Similarly, supportive organizational environment and HR practices seem to increase OCB [59,60]. There is also evidence to suggest that the relationships between group members, cohesive and supportive teams, and perceived support from peers increase OCB [61,62,63]. Effective socialization on entry has also been found to increase OCB [64].
- Skills, traits, and states: Certain personality traits, such as conscientiousness, emotional stability, agreeableness, positive affectivity, intrinsic and extrinsic motivation, and self-esteem have been linked to OCB [63,65,66,67,68,69,70,71,72,73,74]. Opportunities for professional development or training also increase OCB [75]. It is noteworthy that in several studies, well-being and happiness feature at times as an antecedent of OCB rather than an outcome [17,76].
- Job or task characteristics: A variety of task characteristics such as role clarity, task significance, identification with the task, autonomy, and task variety can predict OCB [38,77,78].
- Stress: Several studies [79,80,81,82] reported on stress negatively impacting OCB.
- Work–family conflict: Bolino and Turnley [33] suggested that work–family conflict can reduce employees’ ability to perform OCB due to lack of time or emotional resources. Similar findings were reported by Tompson and Werner [83].

### 1.4. The Negative Consequences of OCB

While OCB has many benefits, it can also lead to some potential negative outcomes for employees and their organizations:

- Overload: Engaging in OCB often results in increased workload [4,5,33], since it involves performing tasks that are beyond one’s formal job requirements.
- Stress and burnout: Since OCB frequently results in increased workload, this, in turn, can lead to stress, depletion of resources, exhaustion, and burnout [5,33,84,85,86,87].
- Citizenship fatigue: Bolino et al. [5] identified citizenship fatigue as a state of feeling overwhelmed and worn out from frequent engagement in OCB. The authors found that it can lead to reduction in subsequent acts of OCB.
- Work–life conflict: Excessive OCB can lead to work–life conflict, as employees may sacrifice their personal time to fulfil these extra-role behaviors. This can lead to increased work–family conflict [34,87,88,89,90] and work–leisure conflict [84,85]. Reich [91] proposed that even when employees are not at work, they are sometimes psychologically connected to their work and may feel guilty about relaxing when they could be working. Lavee and Pindek [84] indicated that in performing OCBs, the lines between work and personal time become blurred and employees may sacrifice breaks and meal time to complete further work-related tasks.
- Role conflict: Exhibiting high OCB can lead to employees experiencing role conflict and a sense of ambiguity in relation to their actual job description [92], which could then further contribute to stress at work.
- Increased expectations: Engaging in OCB may build up expectations from managers and coworkers that the person will continue to engage in these behaviors. As such, the discretionary behaviors may become a part of the employee’s role, leading to potential negative consequences if the behaviors are not maintained [85].
- Risk of exploitation: Employees who frequently exhibit OCB might feel taken for granted or exploited, especially if their efforts are not appropriately acknowledged or rewarded [93].
- Decreased job performance: Researchers have coined the term “OCB-performance paradox” to refer to the notion that, although OCB is associated with increased performance and performance evaluation, it may detract from the time and energy that could be spent on one’s regular or formal job tasks, therefore leading to diminished job performance [4,86].
- OCB as impression management: Bolino [94] suggested that OCB may be displayed as a form of impression management, practiced by “good actors” rather than “good soldiers.” He also made a point that those who exhibit OCB for the propose of impression management may carefully select how and when to perform OCB (e.g., when the supervisor is present and likely to witness it). Grant and Mayer [95] found that some employees may be both good soldiers and good actors. They also found that only the good soldiers engaged in voice behaviors, which is often viewed as a challenging form of OCB, which is less likely to result in favorable feedback from supervisors.
- Deviant or counterproductive behaviors: Several papers reported on a positive association between OCB and counterproductive behaviors [96,97,98,99]. This association has been explained by moral licensing theory [100,101], which suggests that employees who engage in OCB may feel that they have gained “moral credits” that can compensate for counterproductive behavior, hence expecting these to be downplayed or ignored.
- Intent to quit: In several studies, positive correlations have been reported between OCB and turnover intentions [85,90], which can be explained through the relationship between OCB and stress.
- Decreased organizational efficiency: Podsakoff and MacKenzie [39] found that helping behaviors on the part of sales agents actually decreased (rather than increased) agency effectiveness, as measured by a composite sales index.

### 1.5. OCB in Part-Time and Temporary Employees

As noted earlier, there is scarce literature on OCB in part-time or temporary employees [102,103,104,105,106,107,108,109], and to our knowledge, there is only one qualitative [110] and no quantitative research on OCB in part-time working university students.

The research on OCB in part-time or temporary employees draws on a theoretical argument which suggests that employees exhibit OCB as a response to and as a way of reciprocating a consistent, long-term record of positive treatment they receive from the organization [103]. The positive treatment may include direct investment in employees (such as training, promotion, or benefit schemes), the development of social ties, and various rewards. Given that student employees are typically employed on a part-time and temporary basis, the key elements that drives OCB—having a long-term relationship with the organization, and having the opportunity to access training and other benefits—is less likely to be present. Consequently, these employees may view their association with the organization as an economic exchange (whereby employees receive pay in exchange for their contribution), as opposed to a social exchange (in which the exchange is social and psychological, and the boundaries of this exchange are less clearly defined) [103,106].

Findings from several comparative studies [102,104,106,107,109] support this argument, as they found that employees on part-time or fixed-term contracts perform fewer OCBs compared to those with long-term, full-time contracts. However, other studies [105,108] reported that part-time employees performed more OCBs compared to full-time employees. The authors [108] suggested that this could be due to their work status being involuntary. OCBs may be used under these circumstances to enhance employees’ image as a means of keeping their jobs, as well as to increase the chance of being offered a permanent position.

Additional findings from a qualitative study conducted by Scola et al. [110] on student employees whose work status was voluntary, reported that student employees were both willing and highly engaged in OCBs. This suggests that there are other factors, such as organizational identification, satisfaction, and commitment, that might elicit OCBs in part-time and fixed-contract employees.

### 1.6. Part-Time Working University Students

Part-time employment in combination with university studies is becoming increasingly common, and in 2015, over 54% of university students in the UK worked part time [111]. Moreover, part-time work has been described as incompatible with university studies and leisure activities [112]. In part-time working university students, an increase in work hours has been linked to reduced time spent on studying and leisure activities. However, many students choose to sacrifice leisure activities rather than sacrificing study time, leading to a steeper decrease in leisure time [113].

In terms of academic performance, Warren [114] found that part-time employment can lead to lower grades and increased likelihood of dropping out. However, it is important to note that the relationship between part-time work and academic performance may depend on the number of hours worked per week. Light part-time work (less than 20 h per week) does not seem to have a significant negative effect on academic performance [115].

On the positive side, part-time work can enhance students’ time-management skills. Nonis and Hudson [116] found that working students tend to have better time-management skills and are more likely to engage in active learning strategies.

Importantly, part-time work can also provide students with valuable work experience and skills that can enhance their future employability. Broadbridge and Swanson [117] reported that part-time work provides students with important skills and experiences that are attractive to future employers.

### 1.7. The Current Study

In this study, we propose that the context of work investigated in this study—part-time and temporary employment that is conducted alongside undertaking a university course—can influence the ways in which employees engage with OCBs and how these, in turn, impinge on the personal and work outcomes investigated here, namely, well-being, job satisfaction, job stress, and commitment.

The theoretical claims upon which we ground our hypotheses suggest that under conditions of part-time and temporary engagement with work that is seen as supplementing one’s key life goal of completing a university course, and therefore likely to be seen as an economic exchange rather than a social exchange, performing OCBs may in fact result in a mix of positive and negative outcomes, such as increased well-being, work commitment and job satisfaction, but also increased stress, and conflict between work and university work, or work and leisure time.

As noted above, research on OCB in young adults who are engaged in both work and education is very limited, as much of the extant work involved full-time employees. Hence, OCB is not well understood in part-time employees and, in particular, in a student population. In this study, we investigated the relationships between OCB, job stress, work–university conflict, work–leisure conflict, intent to quit, well-being, and job satisfaction in part-time working university students to expand the field of research both in relation to outcomes of OCB in general, and with particular attention given to negative outcomes.

Based on the literature described above, the following hypothesis were formulated:

- **Hypothesis 1:** *OCB will correlate positively with job stress.*
- **Hypothesis 2:** *OCB will correlate positively with work–university conflict and with work–leisure conflict.*
- **Hypothesis 3:** *OCB will negatively correlate with intent to quit.*
- **Hypothesis 4:** *OCB will positively correlate with well-being and with job satisfaction.*
- **Hypothesis 5:** *OCB will positively predict job satisfaction.*
- **Hypothesis 6:** *OCB will positively predict job stress.*
- **Hypothesis 7:** *OCB will positively predict well-being.*

## 2. Methods

The current study is a quantitative study using mainly pre-validated scales to assess the relationships between OCB, job stress, work–university conflict, work–leisure conflict, intent to quit, well-being, and job satisfaction. It also examined whether OCB can predict these key work outcomes. Accordingly, correlation and regression analyses were used to probe these points. This research design was chosen since it can effectively discern the intricate interrelationships between these variables, as well as the predictive capacity of OCB. At the same time, this research design enabled a comparison of the findings to earlier studies that investigated these associations in a similar manner in full-time employees (see for example [8,13,18,25,26,32,34,35]). The chosen design has several benefits. Firstly, correlation provides a straightforward way to determine the strength and direction of linear relationships between two variables [118]. By assessing these relationships, we can begin to identify potential patterns or trends that merit further exploration, which in this study was conducted through the use of regression analyses. Regression analyses, especially multiple regressions, allow for the examination of how multiple predictors alongside the key variable (OCB in the current study) are related to the outcome variable. This can be invaluable in cases such as this, where there may be several interrelated factors influencing the outcome variables [119]. Importantly, multiple regression analysis allows for the control of potential confounding variables, ensuring that the relationships observed are not spurious. This is essential given the multifaceted nature of the factors under study, ensuring that the relationships observed are not merely due to a third variable [119]. As shown in the literature review provided earlier, the interplay between OCB and personal and work outcomes is complex, with some contradictory findings reported in earlier work. By employing regression analyses, it becomes feasible to examine potential mediator or moderator effects, shedding light on more nuanced relationships [118]. This study’s methodological choices align with contemporary practices in organizational psychology and behavioral studies. Utilizing correlations offers a foundational understanding, while regression analyses deepen the investigation, allowing for multifaceted insights into the dynamics of part-time working students and their organizational behaviors.

### 2.1. Participants

The study endeavored to recruit university students (at any university, any study area or level), and the only inclusion criteria in addition to their status as students was that they were employed part time at the time of completing the questionnaires. There was no preference for genders, age, ethnicities, or line of work, and the aim was to recruit participants mainly from the UK and Europe. Data on the students’ subject of study were not collected, though they had to state in which country they attend university.

At the outset, 199 part-time working university students were recruited, but 75 were excluded from the analysis due to incomplete responses, and 2 participants indicating they worked over 40 h per week, which was considered to be full time rather than part time.

The final sample therefore consisted of 122 university students aged 18–50 (M = 21.77, SD = 3.95), of which 62.3% identified as female, 31.15% as male, 4.92% as nonbinary, and 1.64% as other. Most participants (59.02%) described their nationality as British, the second most common nationality was Swedish (6.56%), and the rest (34.42%) were mostly from other European countries. Most participants studied (89.34%) and worked (90.16%) in the UK, and the vast majority (90.16%) were full-time university students and 9.84% part-time students.

### 2.2. Materials

A questionnaire was administered to all participants, containing demographic questions such as questions regarding age, nationality, and employment duration, as well as the following seven scales.

The Organizational Citizenship Behavior Checklist (OCB-C) developed by Fox et al. [120] includes 20 items, and a 5-point Likert-scale ranging from Never to Always. An example item includes: “Please indicate how often, during the past months, you have picked up meal for others at work.” The scale was scored by calculating the mean of all items for each participant. A reliability test carried out showed high reliability (α = 0.89), and the normality test conducted suggested that the data was normally distributed (W = 0.99, *p* = 0.840).

Job stress was measured with a scale created by Motowidlo et al. [121] which includes four items, scored on a 5-point Likert scale (Strongly Disagree to Strongly Agree): for example, “I feel a great deal of stress because of my job.” The scale was scored by calculating the mean for each participant. A reliability test carried showed good reliability (α = 0.78), and the normality test (W = 0.98, *p* = 0.059) suggested that the data was normally distributed.

The work–university conflict scale developed by Lingard [122] included ten items scored on a 7-point Likert scale ranging from Strongly Disagree to Strongly Agree: for example, “The demands of my work interfere with my study.” The scale was scored by calculating the mean. A reliability test showed good reliability (α = 0.89), and the normality test (W = 0.98, *p* = 0.126) suggested that the data was normally distributed. We note that work–university conflict was selected for this study (as opposed to work–family conflict) since, in young adulthood, education and part-time employment are thought to be the main life domains, and the number of hours spent at a place of employment positively correlates to work–university conflict [123].

The work–leisure conflict scale created by Wong and Lin [124] has five items and a 5-point Likert scale ranging from Never to Very Often. An example item includes: “I do not have enough time for leisure activities because of my job.” The score for each participant was calculated by computing the mean. A reliability test showed high reliability (α = 0.89), and the normality test (W = 0.98, *p* = 0.138) suggested that the data was normally distributed.

The Intent to Quit Scale developed by Begley and Czajka [125] consists of two items scored on a 7-point Likert scale that ranged from Strongly Disagree to Strongly Agree, such as “I often think about quitting my job.” The score for each participant was calculated by computing the mean. A reliability test showed good reliability (α = 0.81), and the normality test (W = 0.93, *p* < 0.001) suggested that the data was not normally distributed.

The PERMA-Profiler developed by Butler and Kern [126] is a commonly used well-being measure that consists of 23 items. An example item includes: “In general, how often do you feel sad?” The items are scored on a 10-point scale. Subscales include: Positive Emotion, Engagement, Relationships, Meaning, Accomplishment, Negative Emotion, and Health. Overall, well-being score was calculated as suggested by the authors [126] by calculating the average of 16 key items. A reliability test carried out showed excellent reliability (α = 0.91), but the normality test (W = 0.89, *p* < 0.001) suggested that the data was not normally distributed.

Job satisfaction: A single item drawn from Dolbier et al. [127] was used to measure job satisfaction: “Taking everything into consideration, how do you feel about your job as a whole?” It was scored on a 7-point Likert scale that ranged from Extremely Dissatisfied to Extremely Satisfied.

In the design phase of this study, several steps were taken to control for Common Method Bias (CMB). Firstly, the measures for different constructs were pre-validated and commonly used scales, and these were clearly differentiated to reduce the likelihood of confusion or overlap. Furthermore, respondents were assured strict anonymity and confidentiality of their responses, reducing evaluation apprehension that could lead to distorted responses.

### 2.3. Procedure

Participants who were recruited via social networks were messaged privately using a standardized message either in English or translated into Swedish if Swedish was their native language. A standardized message was also posted on the first author’s social media accounts and advertisements in the form of flyers were put up as posters as well as left in classrooms of a UK university. Participants anonymously completed the questionnaire online after reading through an information sheet and electronically signing a consent form. After completing the questionnaire, participants were debriefed about the aim of the study and offered to sign their email up for a prize draw for a £25 Amazon voucher.

We note that online surveys can be a beneficial method for collecting data on prosocial behavior, but there are also some limitations to this approach. The benefits include a wide reach, cost-effectiveness, anonymity, convenience for participants (which may increase participation rates), and minimization of data entry errors. The limitations include sample bias (dependence on accessibility to the internet or to technology), misinterpretation of questions (that cannot be clarified without the researcher’s presence), lack of control over the environment (where the participant completes the survey), and, importantly, social desirability bias, which tends to occur more frequently in research that requires reporting on one’s behaviors (as is the case in the current paper), as well as lack of depth or nuances [128,129,130,131].

### 2.4. Data Analysis

To test the hypotheses that OCB is positively correlated to job stress, work–university conflict, work–leisure conflict, intent to quit, well-being, and job satisfaction, a Spearman’s rho correlational analysis was performed. The Spearman’s rho correlation analysis was chosen because the assumption of normality was not met, which was examined though a Shapiro–Wilk test and revealed that the data for all scales but intentions to quit and well-being were normally distributed.

The analyses were also corrected using a Bonferroni correction, since a large number of correlations were computed at the same time.

A linear regression analysis was conducted to test the hypothesis that OCB predicts job stress, job satisfaction, and well-being. A sample size calculator was used to ascertain the sample size required for a multiple regression study with 5 predictors at a probability level of 0.05. The result indicated that the required sample size was 93.

Heteroscedasticity and linearity were assessed for all linear models computed and since diagnostic plots indicated possible issues with heteroscedasticity and linearity in the linear model predicting job satisfaction. It was therefore compared to a robust version of the model to examine whether the interpretation of the model would change if it was adjusted for heteroscedasticity. However, the robust model parameters with robust standard errors and confidence intervals were very similar to that of the original model, and the tests for bias were nonsignificant.

There were no outliers or influential cases in the data as there were no standardized residuals with absolute values greater than 3, and the case with the highest Cook’s distance had a Cook’s distance of 0.09.

Moreover, the model parameters were compared to bootstrapped parameters given the possible issues with heteroscedasticity and linearity, but the values were very similar to those of the original model, and since the assumptions checks showed that the original model was not improperly affected by bias, and a robust model did not change the interpretation or the conclusions that could be drawn from the model, the original model was therefore not adjusted for any type of bias.

### 2.5. Ethics

Ethical approval was obtained from the University of Sussex Psychology School Research Ethics Board (ER/ESMJ20/1). Participants gave informed consent before taking part in the study and were debriefed once they had completed the survey.

## 3. Results

The current study employed a correlational analysis to examine the relationship between OCB, job stress, work–university conflict, work–leisure conflict, intent to quit, well-being, and job satisfaction, as well as a linear regression analysis to examine the relationships further. Demographic information such as field of employment and employment duration was also analyzed. The findings are described below, and descriptive statistics of the scales used for the key variables are shown in Table 1.

The average employment duration of participants was 14.78 months (SD = 16.9, Min = 1, Max = 108), and on average, they worked 13.34 h per week (SD = 6.79, Min = 2, Max = 36), suggesting that students were employed both part-time and holding temporary positions. A third (32.79%) of the participants worked in accommodation and food, and 20.49% in wholesale, retail, and repair of motor vehicles. Other fields of employment included education, healthcare, information and communication, and social work.

Table 2 presents the findings of the correlation analysis. The results revealed that OCB correlated positively with work–university conflict and work–leisure conflict. No other correlations were found between OCB and other variables.

Work–university conflict also positively correlated with work–leisure conflict as well as with job stress, and it negatively correlated with job satisfaction. Further correlations that were observed included job satisfaction correlating negatively with work–leisure conflict, intent to quit, and job stress. No other significant correlations were found.

To investigate the relations of the aforementioned variables further, three linear models were computed. Firstly, a model to predict job satisfaction from OCB, job stress, work–university conflict, work–leisure conflict, intent to quit, and well-being as predictors was performed. The findings from the first model (see Table 3) revealed that OCB, work–university conflict, job stress, and intent to quit significantly predicted job satisfaction. The overall predictive power of the model was R^2^ = 0.59, which means that OCB, work–university conflict, job stress, and intent to quit account for 59% of all the variance in job satisfaction. Adjusted R^2^ = 0.57, *F*(4, 101) = 36.08, and *p* < 0.001.

A second model predicting job stress from OCB, job satisfaction, work–university conflict, work–leisure conflict and intent to quit was fitted. The findings revealed that job satisfaction and OCB were the only significant predictors of job stress (see Table 4). R^2^ for the model was 0.18, which means that job satisfaction accounts for 18% of all the variance in job stress. Adjusted R^2^ = 0.17, *F*(2, 105) = 11.8, and *p* < 0.001.

A third model was fitted to predict well-being from OCB, job stress, work–university conflict, work–leisure conflict, intent to quit, and job satisfaction. The results revealed that only work–university conflict was a significant predictor of well-being (see Table 5). R^2^ for the model was 0.09 which means that work–university conflict accounts for 9% of all the variance in well-being. Adjusted R^2^ = 0.09, *F*(1, 105) = 11.8, and *p* < 0.005.

## 4. Discussion

The current study addressed the outcomes of OCB in part-time and contingent working university students, a group of respondents that has been previously scarcely studied in relation to OCB. The results confirmed Hypothesis 2, which hypothesized that OCB would correlate positively with work–university conflict and work–leisure conflict. Hypotheses 1, 3, and 4 regarding OCB correlating positively with job stress, well-being, and job satisfaction, and negatively correlating with intent to quit, were refuted since no significant correlations were found between OCB and job stress, intent to quit, well-being, and job satisfaction. The results also confirmed hypotheses 5 and 6 as OCB positively predicted job satisfaction and job stress. However, Hypothesis 7 was refuted since OCB was not found to predict well-being. The findings and their interpretations are discussed in relation to relevant literature below.

### 4.1. OCB and Job Stress

In many full-time working samples, OCB correlates with stress either negatively [34] or positively [85]. However, no correlation was found between OCB and job stress in the current sample. Nevertheless, OCB predicted job stress together with job satisfaction. A possible explanation for the lack of correlation could be that OCB was not performed excessively in this group (mean = 2.94 on a scale of 1–5), which aligns with earlier research findings [104] and hence did not lead to high stress levels (mean = 3.09 on a scale of 1–5). Nevertheless, when controlling for job satisfaction, OCB predicted stress. This suggests that job satisfaction may mediate the association between OCB and stress. That is, when satisfaction is low, performing OCB can lead to stress, while when job satisfaction is high, performing OCB does not lead to stress. A related point to highlight is that, in this sample, job satisfaction negatively predicted job stress, a finding that is aligned with earlier work [132,133,134]. According to Shin and Jung [132], job stress and job satisfaction are related through a variety of extrinsic and intrinsic factors that influence both variables such as work environment and intrinsic motivation.

### 4.2. OCB, Work–University Conflict and Work–Leisure Conflict

The findings of the current study revealed that OCB correlated positively with work–university conflict and work–leisure conflict. This finding is aligned with the findings of earlier research [34,84,90].

Work–university conflict also positively correlated with job stress in the current sample, similar to earlier research [135]. However, work–leisure conflict did not correlate with job stress, which is inconsistent with other findings in the field [136]. This suggests that while a conflict between work and university work leads to increased stress, work–leisure conflict is perhaps expected to some degree, and since leisure is not subject to externally enforced deadlines, it does not lead to increased stress. In line with this analysis, work–university conflict also correlated in the current sample negatively with job satisfaction, while work–leisure conflict did not, suggesting that work–leisure conflict does not detract from one’s view of their job. Hall [113] claimed that students tend to sacrifice leisure activities before compromising study time. Since many students cannot afford not to work, jeopardizing leisure time may seem to be the obvious sacrifice. Additionally, while university demands tend to be inflexible with set deadlines, leisure time is more flexible [113].

The results also showed a positive correlation between work–university conflict and work–leisure conflict, which fits in well with research on the positive association between work–family conflict and work–leisure conflict [137], suggesting that work–leisure conflict is an extension of work–family conflict.

### 4.3. OCB and Intent to Quit

Opposing Chen et al.’s [46] idea about intent to quit manifesting itself as putting in less effort and thus performing less OCBs, or Bolino et al.’s [85] theory that OCB leads to stress and therefore might cause employees to want to quit, no correlation between OCB and intent to quit was found in the current sample. This aligns with findings from Aryee et al. [138], who did not find a correlation between a subscale of OCB (OCBI) and intent to quit. It has also been claimed that the relationship between OCB and intent to quit differs in full-time and part-time working samples [139,140], suggesting that future research should examine the relationship between OCB and intent to quit further, looking into the factors that influence this association, and why it differs in part-time compared to full-time employees.

### 4.4. OCB and Well-Being

OCB was not found to correlate with well-being in the current sample, which is inconsistent with the literature, most of which shows that OCB (as well as prosocial behavior in general) is positively associated with well-being [15,18,141]. A possible explanation is that this sample is moderately engaged with OCB, hence this level of engagement may not impinge on their well-being.

The only variable that well-being was correlated negatively with was work–university conflict. This is consistent with research on work–family conflict that has reported that work–family conflict tends to lead to reduced well-being [142]. This point provides further evidence that work–university conflict is a good substitute for work–family conflict in university students. It also demonstrates the ways in which increased conflict between two major life domains is associated with poorer well-being.

### 4.5. OCB and Job Satisfaction

Together with work–university conflict, job stress and intent to quit, OCB positively and significantly predicted job satisfaction. This aligns with most evidence from the field suggesting an association between OCB and job satisfaction [19,20,21,57,143].

Additionally, work–university conflict negatively predicted job satisfaction in the current sample, a finding that is in line with earlier research [144,145], and as stated above, job stress has been found to have an inverse relationship with job satisfaction, similar to earlier findings [132,133]. The same is true for job satisfaction, which was negatively predicted in the current study by intent to quit and therefore aligns with earlier work [146]. This shows that, while some relationships investigated by the current study appear to be different in part-time employed university students compared to full-time employees, variables associated with job satisfaction and the direction of these relationships appear to be largely the same as in full-time working samples.

### 4.6. Implications, Limitations and Future Directions

The current study sheds light on consequences of OCB in a population previously understudied, using a measure that has not been extensively researched, namely, work–university conflict. It shows how work–university conflict relates to OCB, work–leisure conflict, job stress, job satisfaction and well-being in part-time working university students. Furthermore, work–university conflict appears to relate to similar variables and in a similar way to those variables as work–family conflict, which suggests that work–university conflict is a good substitute for work–family conflict in university populations. However, research into work–university conflict and its related variables is very limited, and it is important to investigate this phenomenon further to understand how this might affect part-time working university students, both from an organizational perspective and a well-being perspective, since increased work–university conflict is associated with increased job stress, decreased job satisfaction and decreased OCB.

The results show that several well-established relationships in full-time working populations may not occur in part-time working student populations, and the study is the first to examine OCB and its consequences in temporary and part-time working university student sample. This suggests that further research is required both theoretically and empirically to understand the ways in which part-time work, particularly if seen as temporary, impinges on staff willingness to perform and engage with OCB.

The managerial implications that can be drawn from these findings, particularly for sectors that tend to employ part-time students seasonally, is that work–university conflict can result in reduced OCB, which was found to be a strong predictor of job satisfaction and job stress. Additionally, work–university conflict predicts reduced well-being. This suggests that employers need to work proactively to allow students some flexibility during stressful periods (such as exam times), in order to reduce work–university conflict, thereby leading to improved OCB, well-being and job satisfaction.

This study has several limitations. Firstly, given the correlational nature of the study, no conclusions can be drawn regarding causality and generalization may be limited by the fact that the sample largely consists of women and mostly Western students. Therefore, further research should examine the outcomes of OCB in other student samples. Additionally, as mentioned earlier, OCB can be investigated through the use of empirical methods that are more objective than self-report questionnaires and that can observe, record and count the target behaviors. Unfortunately, this study did not have access to such measures.

In sum, part-time working university students seem to experience a role conflict between work and university, and as expected based on research in full-time employees, this impinges on their job satisfaction, stress and well-being and leads to reduced OCB. Moreover, OCBs performed by employees were related to increased work–university conflict and work–leisure conflict, but also predicted increased job satisfaction and job stress. Interestingly, OCB did not correlate with intent to quit or well-being as it is commonly found in full-time working populations. The differences between the current study’s findings and other research, mostly conducted in full-time employees, merit further investigation to gain a better insight on the sources of these differences.

## Figures and Tables

**Table 1:** Descriptive statistics.

	Mean	SD	Scale Range
OCB	2.94	0.67	1–5
Job stress	3.09	0.95	1–5
Work–university conflict	3.62	1.28	1–7
Work–leisure conflict	2.75	0.92	1–5
Job satisfaction	4.94	1.40	1–7
	**Median**	**SD**	
Intent to quit	3.75	1.92	1–7
Well-being	6.41	1.33	1–10

Note: Median was used for non-normally distributed data.

**Table 2:** Correlation coefficients.

Variables	1	2	3	4	5	6	7
1. OCB	-	0.16	0.34 **	0.29 *	0.01	0.2	0.10
2. Job stress	-	-	0.32 *	0.26	0.24	−0.15	−0.32 *
3. Work–university conflict	-	-	-	0.47 ***	0.21	−0.24	−0.32 *
4. Work–leisure conflict	-	-	-	-	0.27	−0.09	−0.32 *
5. Intent to quit	-	-	-	-	-	−0.18	−0.70 ***
6. Well-being	-	-	-	-	-	-	0.29
7. Job satisfaction	-	-	-	-	-	-	-

Note: The correlation coefficients shown are Spearman’s rho coefficients. * *p* < 0.05, ** *p* < 0.005, *** *p* < 0.001.

**Table 3:** Job satisfaction linear model.

Effect	Estimate	SE	95% CI		P
			LL	UL	
Intercept	0.00	0.06	−0.13	0.13	1.00
OCB	0.26	0.07	0.12	0.39	0.00
Work–university conflict	−0.26	0.07	−0.41	−0.12	0.00
Job stress	−0.18	0.07	−0.32	−0.04	0.01
Intent to quit	−0.56	0.07	−0.70	−0.43	0.00

Note: Estimate = Beta coefficient. N = 122. CI = Confidence interval. LL = Lower limit. UL = Upper limit.

**Table 4:** Job stress linear model.

Effect	Estimate	SE	95% CI		P
			LL	UL	
Intercept	0.00	0.09	−0.17	0.17	1.00
OCB	0.23	0.09	0.06	0.41	0.01
Job satisfaction	−0.39	0.09	−0.57	−0.21	0.00

Note: Estimate = Beta coefficient. N = 122. CI = Confidence interval. LL = Lower limit. UL = Upper limit.

**Table 5:** Well-being linear model.

Effect	Estimate	SE	95% CI		P
			LL	UL	
Intercept	−0.007	0.092	−0.19	0.18	0.001
Work–university conflict	−0.30	0.12	−0.54	−0.06	0.01

Note: Estimate = Beta coefficient. N = 122. CI = Confidence interval. LL = Lower limit. UL = Upper limit.

## Data Availability

Data collected for the purpose of this study can be obtained from the authors upon request.
